# When Personal Tracking Becomes Social: Examining the Use of Instagram for Healthy Eating

**DOI:** 10.1145/3025453.3025747

**Published:** 2017-05-02

**Authors:** Chia-Fang Chung, Elena Agapie, Jessica Schroeder, Sonali Mishra, James Fogarty, Sean A. Munson

**Affiliations:** 1 Human Centered Design & Engineering, University of Washington; 2 Computer Science & Engineering, University of Washington; 3 The Information School DUB Group, University of Washington

**Keywords:** Self-Tracking, Personal Informatics, Social Support, Health, Social Media, Food Journals, H5.m. Information interfaces and presentation (e.g., HCI)

## Abstract

Many people appropriate social media and online communities in their pursuit of personal health goals, such as healthy eating or increased physical activity. However, people struggle with impression management, and with reaching the right audiences when they share health information on these platforms. Instagram, a popular photo-based social media platform, has attracted many people who post and share their food photos. We aim to inform the design of tools to support healthy behaviors by understanding how people appropriate Instagram to track and share food data, the benefits they obtain from doing so, and the challenges they encounter. We interviewed 16 women who consistently record and share what they eat on Instagram. Participants tracked to support themselves and others in their pursuit of healthy eating goals. They sought social support for their own tracking and healthy behaviors and strove to provide that support for others. People adapted their personal tracking practices to better receive and give this support. Applying these results to the design of health tracking tools has the potential to help people better access social support.

## Introduction

Many people use social media and online communities to support healthy behavior change [31] and chronic disease management [43,53]. Some seek emotional support, accountability, motivation, and information from others on these platforms [43]. Others keep track of illness-related symptoms, hoping these data could lead to better diagnoses and treatments [53]. However, user concern about how health information may affect their image influences what and with whom they are comfortable sharing.

Since the launch of Instagram in 2010, the mobile photo- and video-based social media app has rapidly increased its user base to over 600 million people [47]. Food photos are particularly prevalent [37], and the popularity of food photos has drawn attention from HCI and CSCW researchers. Using Instagram as a food photo database [38], researchers were able to identify food deserts [11] and associate eating habits with locations [36]. Researchers have also examined how the platform is used to promote eating disorder behavior [9,45].

Some people post food photos to Instagram to support their health goals, using it as an everyday tracking tool as they pursue healthy eating choices. Understanding why and how people appropriate Instagram to track and share food data has the potential to inform system design to support healthy eating. We look at people's practices through different stages of tracking: their choice of Instagram among other tracking tools, their tracking and reflection practices, and their lapses of tracking. To answer the following research questions, we conducted an interview study with 16 people who frequently record and share what they eat on Instagram:

- Why and how do people keep track of their food on Instagram to support their health goals?
- How does social interaction affect their health tracking decisions and tracking behavior on Instagram?

We found that people track their food on Instagram with a mix of co-existing goals: recording what they eat, receiving social support, and providing social support. We use the lived informatics model [21] as a lens to examine different phases participants experience regarding tracking and sharing what they eat on Instagram. The lived informatics model extends other personal informatics models (e.g., [29]) to describe the decision to track, tool selection, and lapsing and resumption of tracking. Using this model supported our analysis of how and why participants choose Instagram for food tracking among other tools; how they iteratively track and act on these food posts (collecting data, integrating it, and reflecting on it); and how they intentionally or unintentionally lapse and resume tracking. We found that participants adapt their personal tracking behavior in every tracking stage to receive and provide better social support.

Building upon prior work on health information sharing in online communities, we discuss how participants use Instagram features to help receive social support while maintaining their needs for impression management. We also reflect on these findings to discuss how social needs could be integrated into personal informatics models and inform the design of future health tracking tools.

## Background

Our study of how people appropriate Instagram to track and share their food was informed by prior research on technologies to support healthy eating and how people share their health tracking data with other people. To situate sharing of food posts on Instagram within broader research on online health information sharing, we also draw upon prior research on health information sharing in online communities.

### Self-tracking and Personal Informatics Models

People use a myriad of self-tracking tools to collect and reflect on their personal information. To understand how people use these tools and the information they collect, Li et al. developed the five-staged personal informatics model [29]: *preparation*, *collection*, *integration*, *reflection*, and *action*. They later expanded the reflection stage to *maintenance* and *discovery* phases [30].

Rooksby et al. proposed “lived informatics” to describe the intertwined roles of self-tracking plays in people's everyday life [49]. They also described various goals people have while adopting self-tracking practices, such as behavior change, curiosity, or instrumental purposes (having a record, receiving incentives, and maintaining a relationship). Epstein et al. later developed the lived informatics model [21] to better describe people's tool selection choices, the concurrent and iterative nature of collection, integration, and reflection, as well as the lapse or resumption of tracking. The lived informatics model has three major stages: (1) *Deciding and selecting*: In this stage, people make decisions about tracking and choose the tools they want to use. (2) *Tracking and acting*: People *collect* data about themselves, *integrate* that data (sometimes across several tools or data sources), and *reflect* on the data they collected. (3) *Lapsing and resuming*: People periodically forget, intentionally skip, or suspend tracking.

Prior studies of self-tracking to support healthy eating goals have examined ways to ease the burden of *collection* and to include social features to encourage *collection*, *reflection* and *action*. In this study, we provide an in-depth understanding of how people appropriate social media for personal tracking. Our findings add to the current personal informatics models and inform various ways social needs influence personal tracking decisions and practices.

### Technologies to Support Healthy Eating Goals

Traditional methods for tracking food to support healthy eating have relied on associating ingredients with calories (e.g., MyFitnessPal); tracking food with app-computed scores (e.g., Weight Watchers Smart Points, a food scoring system used by Weight Watchers based on calories, saturated fat, sugar, and protein); or scanning food barcodes to avoid the need to enter ingredients (e.g., the Weight Watchers app). These food journals usually rely on food databases, which are not comprehensive: they usually only contain common food items and recipes, and they do not account for variability of ingredients in the actual food people eat [17]. Furthermore, people using such databases often start eating more packaged food, because it is relatively easy to track foods with barcodes or that are otherwise mass-produced. This nudge is often at odds with their healthy eating goals. Even if a comprehensive database existed, item-by-item tracking is tedious and difficult to perform in certain social contexts, which makes it difficult to develop a reliable, long-term habit of food tracking.

Researchers have investigated approaches to enhance the tracking experience and address these issues, such as making tracking easier and more engaging through photo-based journals [16,35], participating in food challenges [20], recording voice memos of food experiences [23], or automatically taking photos of food [4,51]. These lightweight journaling methods can support people's health goals and increase their awareness of their eating habits, including unhealthy eating [6,16,42,55]. In particular, photo-based food journals make it easier to collect data, assess progress, and track failures such as unintended larger meals [16]. However, photo-based tracking also generally means a lack of caloric data relevant to many health goals, and many people still forget to track their food. In a similar tradeoff, fully automated solutions can make information capture easier, but they have also been found to reduce the mindfulness that people gain from food tracking [54].

Previous food tracking systems have often been designed to help people improve their behaviors. Researchers have also called for more research and design focusing on positive interactions and experiences with food, such as aspects of family connectedness, food as gifting, creativity, and pleasure or nostalgia in creating food [24].

### Health Tracking with Social Sharing

Prior research has explored how social features can help engage people with self-tracking technologies and encourage behavior change [32,46,48]. The addition of social features can help users consistently meet their health goals [6,15], can help people receive support through social interactions, and can encourage a feeling of accountability to others [18]. People also often value understanding other's emotions in relation to their food experiences [23].

Baumer et al. introduced *VERA*, a photo-based system in which a user posted a picture of a “health-related” decision they made and others could comment [5]. By leaving the definition of “healthy” to each user's interpretation, people could decide for themselves what constituted a healthy lifestyle. In a follow-up study, they found that users who interacted with others who shared their goals stayed more engaged on VERA [2]. *Community Mosaic*, another photo-based system, allowed users to send messages of food photos to a local community server with explicit goals to help community members improve their healthy diet practice. These photos were later shared anonymously in a public kiosk where other community members could rate whether the photo was inspirational [44]. By thinking about how to provide helpful food photos, users reflect on their own food choices and preparation processes.

Although social features can motivate people to use self-tracking technologies, they can also introduce impression management concerns for their users [15], especially when the tracking technology appropriates existing social media platforms. In an online experiment, automatically posting announcements of their weekly physical activity goals on Facebook decreased people's willingness to set goals [40]. Furthermore, systems that allow people to share their progress on Facebook can cause anxiety about cluttering friend news feeds or appearing boring [41].

### Sharing Health Information in Online Health Communities

According to a 2013 survey, 72% of US internet users search for health information online [22]. Many people also turn to online groups for social support as they pursue lifestyle change goals [31] or manage illnesses [27]. People find guidance and motivation in the day-to-day experiences of people with similar health challenges or similar health goals [13,26,27,31]. They also pursue emotional support in these online health communities. People were able to find and develop a strong sense of community with people who had similar experiences [28,33,34] and receive emotional support to help them move through difficult recoveries or struggles to adopt lifestyle changes [26,31,43].

People have appropriated social media, such as Facebook and Twitter, for searching, sharing health information, and seeking social support from pre-existing relationships [11,43]. People with potentially stigmatized illnesses, such as anorexia or depression, approach Tumblr and Instagram for recovery support [8]. Although these sites can help people struggling with such illnesses, they can also introduce new challenges, such as exposure to pro-anorexic sentiment [8] or negative comments [3]. People trade off these concerns and benefits, along with the needs for impression management [43], to decide where and with whom they share health information.

We studied food journaling on Instagram to understand how people combine photo-based food journaling and social interactions in public Instagram communities to support their healthy eating goals. We found people chose Instagram as a self-tracking tool because of opportunities to both receive and provide social support. They also weigh tradeoffs between consistent tracking, social support, and impression management to make ongoing decisions about what to track, whether and how to act, and when to lapse.

## Methods

We conducted semi-structured interviews with 16 participants who self-identify as using Instagram to support their healthy eating and/or fitness goals. We recruited participants through a pre-screening survey shared via researcher social networks, direct messages to Instagram users who use specific food tracking hashtags (#fooddiary and #foodjournal) and use English in their posts, and snowball sampling via interview participants. Each interview participant was compensated with a $10 gift card. Participants were primarily located in the US, with two from the UK (I09 and I11, Table1).

We asked participants why they decided to use Instagram to pursue their health goals and what their Instagram practices and experiences were regarding healthy eating. We also asked whether and how they interacted with other Instagram users, as well as what role Instagram played in supporting their health goals in relation to other self-tracking tools, social media, and online communities. The interviews lasted 45 to 77 (*M* = 60) minutes. All interviews were audio-recorded and transcribed for further analysis.

We adopted a mix of inductive and deductive approaches to analyze the data. Four researchers first each coded two transcripts and discussed the emergent codes, organizing the codes via affinity diagramming and creating a code book based on our original research questions, the lived informatics model [21], and a prior study on adopting online communities and social media to support health [43].

Prior research on food journaling and other personal informatics domains has shown that tool selection is important for later tracking and action; that data collection, reflection, and action are often concurrent or iterative; and that both short term lapses and abandonment are common. We chose to use the lived informatics model to inform our analysis because it best captures these characteristics.

People share health information in online health communities to seek emotional support, motivation, accountability, and information [43]. We analyzed our data based on these social support needs to understand whether and how they influence participants in their choice to use Instagram and in their tracking and sharing behaviors on Instagram.

We then recoded the same two transcripts using the code book, augmenting with codes that emerged through the process. After discussion, the code book was updated with the emerging codes and used to code the remaining transcripts.

## Why and how do people track what they eat on instagram to support healthy eating?

Our participants use Instagram because it allows them to keep a record of activities relevant to their health goals, while obtaining and providing social support and information to communities with which they identify. People primarily record what they eat and cook. We use the lived informatics model [21] to describe personal tracking in stages. Understanding why people use Instagram for healthy eating goals and how they use Instagram in each of these stages can provide insights about people's evolving goals and unmet needs. Because reflection happened both concurrently with collection and integration (as suggested by the lived informatics model) and on its own at a later time, we discuss reflection separately from collection and integration.

Through the lens of the lived informatics model, we describe health, instrumental, and social-support goals of participants. Many participants sought to improve their health behaviors, while others tracked for instrumental goals such as building an audience or educating others. These goals were not mutually exclusive. Some participants also sought social support through their tracking behavior. We relate the pursuit of receiving and providing social benefits to the type of tracking activities participants perform. We discuss how these themes manifest in the different stages of the lived informatics model.

### Deciding to Track and Selecting Tools

The lived informatics model suggests people select tracking tools based on features, aesthetics, and conveniences [21]. Our participants chose to use Instagram because it provided an easier, more fun, and more social way to track what they eat. Contrary to the individual focus of the lived informatics model, they also often wanted to motivate and encourage others to maintain their health. Many participants had experience with or were concurrently using other tools to record calories and nutrient details in addition to recording food photos on Instagram, such as *MyFitnessPal* (I01, I02, I05, I06, I07, I09, I11, I14), the official *Weight Watchers* app (I06, I16), *iTrackBites* (I15), or *YouFood* (I15). Others also used *Fitbit* (I04, I07, I08, I13) or *Polar watch* (I12, I14) to track their physical activities and posted screenshots or photos of those activities on Instagram.

#### Photo-based food tracking is perceived as fun

Participants considered tracking and sharing food photos on Instagram as an engaging experience, which helped them sustain their interests and keep a record of what they ate. Food photos feel closer to people's experiences than text descriptions: “*I think the fact that Instagram is much more visually stimulating [than MyFitnessPal], really makes the people more real, or it makes the goals more real.*” (I07). I05 described how this increased engagement helped her maintain food journaling not just on Instagram, but also on MyFitnessPal: “*I have a streak on MyFitnessPal since February (over five months at the time of interview). Before I would just use MyFitnessPal for maybe two weeks and then be like, this is too hard and I stop. … It's really helpful to have all the photos because if I forget to enter it in or if I'm busy during the day, I can enter it at the end of the day and I have an accurate log of what I ate.*”

#### Photo-based food tracking is perceived as socially appropriate

Consistent with prior evaluations of mobile-based food journaling [16], participants considered using photos to record what they eat easier and more socially appropriate than a traditional mobile food journal: “*If I was out with friends or something, then a quick snapshot of the food would be easier than saying, ‘Hold on guys, I need to pull up MyFitnessPal and put everything down and the right serving size’*” (I07). As a result, some participants believed tracking with photos better supported their health goals because they could easily take a photo whenever they ate rather than entering all food entries at the end of the day or when they are free: *“[I'd rather] post frequently throughout the day and have short little posts [on Instagram] than do big long posts and do them once a day [using other tools]*” (I15).

#### Instagram helps people find community support

Many participants chose Instagram for the social support they can gain through the many people using Instagram for healthy eating goals. Participants discovered these communities through prior experiences using Instagram for general use (I01, I02, I03, I04, I07, I09, I11), friend recommendations (I05, I12), or other health communities such as Weight Watchers (I06, I10, I15, I16) or Bikini Body Guide (I08, I13, I14). Many participants found Instagram users more supportive than users of other social media platforms. For example, I05 did not think Facebook was a proper place to share her everyday diet and workout: “*A lot of my friends on social media are acquaintances or old coworkers … they have some connection to you but not enough so that they care about actually supporting you*.”

To find communities, participants often augmented their posts with hashtags. They decided which hashtags to use based on other people's posts or popular food hashtags from Instagram's autofill features. They also learned new hashtags by noting ones that were used concurrently with the ones they knew. After determining whether the hashtag caught the attention of people they wanted to attract (e.g., people belonging to specific communities, such as #weightwatchers), they gradually settled on a fixed set of hashtags used for most of their posts. I07 explained: “*I think the hashtags [help] us find each other …. If I was just posting these photos to an empty wall, there was no way that I'd be connected to others with similar goals to me.*” Hashtags are especially useful for people following a specific program or participating in an event. I08 met other women participating in the same health challenge through hashtags (e.g., #oneperfectweek) and would use such tags to find updates from others at a similar stage of progress. Many participants also use hashtags to find people across communities. For example, participants who investigated hashtags like “#caloriecounting” or “#weightloss” often encountered Instagrammers who subscribed to alternate diet plans. Through this exploration, they discovered different diet and exercise routines.

#### Instagram allows people to motivate and educate others

Some participants chose Instagram because it gave them a way to share their experience with, motivate, and educate other people. I06 mentioned that she started posting food and workouts on Instagram because she wanted her weight loss experience to motivate others: *“Like people that are trying to lose weight and get healthy and might need some extra motivation or just feel stuck or maybe discouraged. I had hoped they would find me, and I could help inspire and motivate them in some way.*” Similarly, I04 started to share her cooking on Instagram to help friends who wanted to improve their cooking skills to eat healthier. These cooking posts often led to comments, messages, and even phone calls from friends wanting food preparation details: “‘*What flour did you use, and how did you get it? Can you come and teach me?’ Then we just had this discussion about the same thing or maybe you should try it this way*” (I04).

### Collection and Integration

In our analysis, *collection* occurs when participants post about their food or fitness on Instagram. We define *integration* as incorporating other information related to their health goals in the photos or captions. The lived informatics model suggests people collect and integrate data differently depending on their motivation for tracking. For example, people who track to change their behavior usually track frequently, while instrumental trackers collect data when the benefits outweigh the costs. Participants also integrate data when integration generates insight. Our participants tracked for a mix of behavior change and instrumental goals. Receiving and providing social support motivated them to collect data consistently, and it sometimes motivated them to integrate data from multiple tracking tools.

#### Accountability: Tracking to keep one and others accountable

Participants frequently said that Instagram supported their health goals by giving them a sense of accountability. Social accountability refers to sharing one's progress to support their continued pursuit of their health goals [35]. Participants described collecting and integrating data on Instagram to stay accountable to themselves and others.

Participants felt that sharing their food photos and having people follow their posts helped them remain accountable for their tracking and health goals. For this reason, participants described feeling a need to honestly record everything: “*I'd post everything even if that's not super healthy*” (I06). I07 would sometimes ‘cheat’ on MyFitnessPal by leaving out small treats, but would post everything on Instagram, which held her accountable to her goal: “*before [when using MyFitnessPal] I would have a small snack pack that was a bag of chips and be like, ‘Oh, that doesn't really count because it's just a little tiny bag.’ But I think with Instagram, it helped me because I was taking a picture of it - It's real and it exists and it does count towards what I was eating. And then putting a visual image of it up really helped me stay honest*.” This accountability is important because missing entries commonly results in abandonment [17].

#### Emotional Support: Sharing tracked data for support

Participants posted and shared what they ate to receive emotional support. By finding others interested in similar diet options, having the same goals, or going through the same weight loss processes, many participants felt better supported by Instagram than by their friends and families: “*It makes me feel like I'm then around other people that feel the same, that have the same goals as me, and it can support me in that; whereas I don't particularly have that with my friends and family just because they're not particularly interested anymore*” (I09).

Participants often posted particularly difficult moments, hoping to receive emotional support. Participants generally perceived Instagram users as being open about sharing bad days and encouraging to each other: “*people are acknowledging that I'm trying*” (I02) and “*there were people rooting for me*.” (I15). Sharing failures (i.e., behaviors conflicting with the individual's health goals) helped them get the emotional support they needed to accept their failures and move on. I15 described a motivational quote she posted when she had a bad day: “*Some of my followers, and people who I follow as well, commented and wished me well and sent me love. It didn't make my day any better but it did make me feel better about everything.*”

Participants also used captions to attract support from others by helping others relate to their experience. Over time, I01 found that adding more personal messages in captions helps her to get more reactions from followers: “*To be a little less objective, a little more personal, to share more of what I'm feeling versus just stating [in the caption]. … I find that people respond better to that. People will comment more. It almost feels as if they're encouraging me because people want to support each other*.”

Participants strove to make the photos they posted visually appealing because they felt that more attractive photos get more attention from other Instagram users. This attention helped increase their motivation for posting and sharing: “*I notice that when I had something that looked tasty and healthy, I would get more people liking the photo… I think I really used that as motivation too*” (I07). Participants had various strategies for taking proper food photos. They would adjust layout and lighting or use various filters or third-party apps. These strategies sometimes affected their food choices: “*[I started to make and eat more vegetarian food] because I think a lot of vegetarian food and vegan food that I make has a lot of green stuff in it and it's very attractive to people*” (I05). Participants also considered timing of posts strategically to get social interactions from other users: “*I've kind of figured out certain times during the day that more people seem to be active on Instagram*” (I03).

#### Motivation: Tracking to motivate oneself and others

Participants posted about accomplishments and experiences to motivate themselves and others. Some participants posted accomplishments, such as reaching weight or fitness goals. For example, I06 posted a day when she reached a large number of steps: “*I had a really good day, and I had gotten 16,000 or more steps, then I feel like I want to share my progress and everything with everyone*.” (I06).

Others posted on Instagram to provide motivation to others. I06 thought that “*show[ing] others what I was going through and eating and everything would help inspire and motivate them*.” I15 mentioned that she kept posting her food even after she reached her goal weight because she wanted to become a “*source of motivation and inspiration for people;*” she thought that continuing to share her previous and current habits would help encourage others.

#### Information: Posting to share information and ideas

Participants also commonly posted what they eat on Instagram to share knowledge about and ideas for healthy eating to others. For example, I01 wanted to convey that dieting does not require giving up flavorful food: “*I just wanted to show people that you can eat food that tastes good and still meets it. It has all the protein and the carbohydrates and the healthy fats, I still hit within the caloric range that I'm aiming for, but it tastes good. It doesn't feel like I'm eating broccoli and chicken*” (I01).

To make food posts useful for others, participants supplemented food photos with informative comments and hashtags. Many participants included recipes and food preparation information in their posts. Since it is difficult to include URLs in comments on Instagram, participants posted full recipes inside the comment instead. Some used hashtags to represent the ingredients to make it easier for others to search for the recipe: “*If I used a particular ingredient or method, I would sometimes use that as a hashtag. An example of that is, you can make kind of fake spaghetti noodles out of zucchini using a spiralizer and they're called zoodles, so I would use the hashtag for zoodles if I made something with that*” (I13).

Some participants integrated information from other tools into their food posts to provide more details. I11 shared her Instagram posts on Tumblr, where she could include more details, and provided the Tumblr link in her Instagram profile: “*If I post a picture on Instagram and then they go to the blog to see the entire recipe then they can actually click on the links*.” I06 would calculate Smart Points and add that to her photos: “*It helps because a lot of people that might be using Weight Watchers usually are saying like how many Smart Points is in whatever they are eating. …That helps when you see people posting stuff like that so then you can kind of get ideas on what to eat.*” Others also often manually incorporated caloric information in their text descriptions, hashtags (e.g., #200calories), or photos using photo-editing tools to help others and themselves reflect and search for previously cooked foods based on calories.

Participants also considered what post content and timings would be most appropriate and useful for their followers. For example, I01 would deliberately make variations of the same food or perform easier workouts so others can follow more easily. I11 once wanted to share a coffee with sugar-free syrup, but decided to post at a time when the content would be more appropriate for her followers: “*I took the picture and then I was like, ‘Is it good to post a picture with coffee at 10:00 in the evening UK?’*”

### Reflection

Participants often reflected on their decisions of eating and tracking by reading their old posts and by reading and commenting on other people's posts. The lived informatics model suggests that people who track to change their behavior usually review their data more often than instrumental trackers. Our participants saw reflection as a way to receive and provide social support, and thus were motivated to reflect on their data and that of others.

#### Accountability: Reflecting on old posts to stay accountable

Participants maintained accountability by reviewing and reflecting on what they had done in the past. Participants found that looking back at their posts made them aware of how well they were maintaining their desired diet and helped keep them accountable. One participant noted how much she had eaten out in the past week and decided to cook more meals at home: *“if I've posted a lot of pictures of food from restaurants, it also kind of tells me, ‘Hey, you haven't had anything that's been homemade in a while.* You've been eating out a lot. Maybe you should make a salad for yourself.’” (I03). Another participant decided to pack her lunches at night after noticing she was eating out too much: “One trend that I've noticed is that I definitely need to take the time to pack a lunch at night before work because if I don't … I'm going to eat a burrito … I'll take a photo with a burrito in my hand in the car. It's helpful for me to see that and look back at the different days” (I13).

Participants also checked other people's posts to keep others accountable to their goals. I08 mentioned she checked in with other women participating in the same healthy eating challenge to make sure they are making progress: “*We are finishing up day 2 of this one perfect week so I'm just checking in, seeing how they are doing and asking how their day went. This is also a photo to show like, ‘Oh, this is what I had for dinner.’ I tagged them and then asked for them to tag me in any of their photos.*”

#### Emotional Support: Reading and commenting for support

Participants read comments from other people and reviewed the ‘likes’ their posts received to gain emotional support. I01 described what kind of feedback she got and how she was encouraged: *“People will send a combination of emoticons in kind of encouragement. You'll see people that say ‘nice’, or ‘good job’, ‘keep it up’. You'll have like clapping hands, you'll have smiley faces or thumbs up kind of thing. It's really fun to have that kind of nice little, even emoji feedbacks, it's super positive for me*.”

Participants also felt that giving emotional support back to the community was important. To provide this support, they proactively looked for other people's posts to comment on. They commented on posts by their usual followers, people they follow, and people who use the same hashtags: “*I'll still go to these certain hashtags and scroll through and comment and encourage other people on some posts if they need encouragement or anything like that … not just ‘like’ other pictures just to get my Instagram handle out there but to actually add to the community*” (I14).

#### Motivation: Reading and commenting for motivation

Participants found looking back at their feed motivating, particularly during weeks when they were not performing as well towards their goal: “*I felt like I'm stuck, and I wasn't losing weight or anything. I had a bad couple of weeks, so I wanted to go back and see what was I eating a few weeks ago where I was feeling really good and doing good just to refresh my mind and re-motivate myself*” (I06).

Seeing posts from other people was also inspiring and motivating. Many participants, such as I12, described being motivated by seeing what others do: “*There's this picture [another Instagram user] posted today: ‘Look at me doing tuck jumps. I couldn't do tuck jumps when I started.’ It probably would have killed her. She can do it and I see her … It's like, ‘I guess I can do it.’*”

People also found motivation by motivating others. I03 described how she commented on posts from other women struggling with eating disorder recovery: “*I want to motivate those other girls that are struggling to say, ‘Hey, I'm in recovery, too, and look what I'm eating.’ Like, I've had to go over the same kind of fears, of different fear-foods and stuff. I kind of use it as more of a motivation*.”

#### Information: Reviewing posts for inspiration and information

To help make day-to-day decisions that support their health goals and to find inspiration, participants often looked for ideas and information in their old posts. Participants browsed old posts to find ideas about what to eat or make: “*if I'm wondering what I should make for the week, I like looking through my posts to see other things I cooked before*” (I07). This reflection was particularly useful when they wanted to replicate previous successes: “*when I hit a plateau, going back and seeing, ‘Oh, I remember that that week there was a sudden loss. Let's go and see exactly what I ate.’ It's very easy to track and replicate*” (I11).

Reviewing other people's food posts also helped our participants obtain ideas and knowledge about food, which was useful to sustain their eating behavior change. As I06 described, new ideas kept her motivated about maintaining her diet goal: “*if I get kind of bored with stuff I'm eating, I can kind of look and see maybe what other people are eating to give me some of the ideas.* “

### Lapsing and Resuming Tracking

The lived informatics model categorizes four types of lapsing: forgetting, upkeep, skipping, and suspending. Our participants did not report lapsing because of forgetting or upkeep. They did sometimes intentionally skip or suspend tracking for reasons related to themselves and others. They often received social support when they lapsed.

#### Accountability: Lapsing when accountability needs change

Participants often lapsed when their goals shifted and caused their accountability needs to change. Some participants were rigorous about tracking when they were trying to change their behavior but relaxed when they were satisfied with it (I03, I04, I07). I03 described how her level of tracking loosened when she reached a goal: *“I'm on a vacation, then I eat out* … *Suddenly I come back on Monday. I eat at home the whole time because I have to make up for eating out. ‘I'm going to keep track until Friday,’ and then I'm like, ‘Okay, so now I'm back on track, so I don't have to keep tracking anymore.’*”

Other participants lapsed because they wanted to help others keep accountable to their goals and worried that certain posts could negatively affect the behavior of their followers. I13 decided that she would not post certain food photos that could cause negative behavior in others: “*I just don't want to post too many potentially triggering photos because they have no way of opting out of seeing them.*”

The goal of accountability was also sometimes at odds with impression management, and participants sometimes lapsed to preserve their image. For example, some were reluctant to post food perceived as undesirable, and I04 worried about her parents seeing posts that showed her drinking: “*If I take pictures of something that I've been drinking, I don't share it with my parents because they don't like me drinking … that's to share with friends*” (I04).

Alternatively, some participants felt uncomfortable lapsing because of their perceived accountability to others. They felt the need to explain their behavior because they did not want to disappoint others. I09 described one instance when she went traveling and did not have a reliable internet connection for three days. Concerned that her followers were waiting for her posts, she explained her connectivity issues to her followers in her first post after her return. I11 similarly felt that her followers were *“looking at [her] as the UK keto guide”.* Because of this perceived accountability, she felt she needed to justify actions that did not befit a role model: “*why I’ve eaten bad that day*.”

#### Emotional Support: Lapsing triggered additional support

Participants mentioned that their lapses sometimes lead to additional emotional support from others, which could help them resume tracking. After being sick and not posting on Instagram for a while, I12 found others left encouraging comments when she resumed posting: “*The girls are like, ‘Yeah, see that's good. You're still eating healthy. At least you're doing it.’*” Some participants even received in-app messages from others when they had not posted for a while (I06, I10, I12): *“I had someone message me, and I had no idea who it was, and she had like commented in some of my pictures, and she messaged me to see me if I'm okay because I hadn't posted anything in a couple of days, and I was like ‘Wow.’ I'm like ‘Thanks.’* … *it made me feel good and kind of got me motivated that like someone that I don't even know takes the time to send me a message to make sure everything was okay”* (I06). These comments and messages made participants feel they were cared for and supported, with messages helping them go through difficult time and resume their everyday tracking activities.

#### Information: Lapsing when photos lacked useful information

Participants whose primary goal on Instagram was to provide knowledge to others were more selective about their posts, intentionally posting about food or workouts to be informative rather than posting all the food they ate or exercises they did (I04, I11, I15). For example, I04 only shared food she had not previously posted because she did not think reposting the same food would be useful for other people: *“I will post maybe once a week or twice a week. Then I won't repeat my posts. I wouldn't post something that I already posted* … *I post something that's interesting*.”

## Discussion

People track their food on Instagram with the intertwined goals of supporting healthy eating and providing and receiving social support. The lived informatics model and other personal informatics models may be strengthened by the inclusion of social interaction and other social roles in the model. As seen among our participants, use of Instagram for food journaling can support both health and social goals, but this use requires people to make tradeoffs between consistent tracking and social needs. Designs can better support people in making these decisions.

### Including Social Roles in Personal Informatics Models

Our findings show that people's interactions with others can influence personal tracking behaviors. Prior research established that others, such as health care providers [12,35] or family members [25,50], influence people's tracking and health decisions and can help them maintain their health. Involvement of others can offer accountability and guidance on how to track as well as how to interpret and act on tracked data. Although the importance of others in health behaviors and in self-tracking decisions is well-established, the models the HCI community uses to understand and design for self-tracking tools [21,29] still focus on the individual.

Our study of Instagram for food tracking offers an in-depth understanding of the many ways others can influence tracking decisions and related behaviors. Participants chose to use Instagram in their food tracking for many of the same reasons people share health information in other forums: to receive accountability, motivation, emotional support, and information from others. But they also chose to use, or continue using, Instagram for their food tracking so they could offer this social support to others.

This motivation carried through to the collection stage of tracking. Our participants created and curated posts to make them desirable, motivating, inspirational, and informative for themselves and others. Our participants also reflected on data collected both by themselves and others, which differs from the emphasis of personal informatics on reflecting primarily on one's own data. Prior work has demonstrated that reflecting on family member's tracking data [50] and receiving prompts from health experts [35] can address challenges that arise in reflection.

The desire to pursue or provide social support can also have negative consequences, triggering lapsing and resumption in tracking. For example, only posting informative photos can leave tracking data incomplete for later reflection on behavior change goals. However, people might have other goals for reflection, and some recent studies (e.g., [19]) have begun to examine how to make incomplete tracking data useful to support such reflection. By understanding why people lapse and resume, and how these reasons conflict with their goals for reflection, systems can also make people aware of these decisions. Systems can also help people make informed decisions that accommodate tradeoffs between social needs and reflection, such as the frequency or the type of posts.

Updating personal informatics models to more explicitly help designers and researchers consider the different roles and influences people may have in self-tracking could lead to new design opportunities. Below, we discuss some of those opportunities in the food tracking domain.

### Using Hashtags to Reveal Myriad Communities

Previous work has suggested that hashtags can induce a sense of ad hoc community [3]. Our findings suggest that this function of hashtags was an important reason users select Instagram for food tracking. The ability to use multiple hashtags allowed users to easily find similar others and to discover and enter new communities. This exposure to new communities helped them find ideas for new exercise and diet options (e.g., new ingredient options to try) and develop their own recipes or exercise regimen.

Prior work in online communities [3,31,43] and health tracking [14,52] has shown the value of social support from similar others. Most online communities and social media platforms also provide features to support people in finding others with the same interests or goals, such as discussion boards with specific topics, Facebook groups, or subreddits. However, commenters with different conditions or illnesses can still share related experiences and provide emotional support [28]. Similarly, strangers on online communities can also provide accountability and information support [43]. Our findings confirm that both similar others and people who are quite different can provide support. Our participants were inspired by users who shared similar healthy eating goals but whose lives were quite different from their own (e.g., students vs. stay-at-home moms). They could also explore new ingredients that are different from their usual choices, but still within their diet restrictions or preferences (e.g., fresh food vs. artificial food substitutes).

However, in Instagram's current form, user discovery of new hashtags depends on serendipity. To better support this type of exploration, systems can show users commonly co-occurring hashtags. Systems can also help users navigate through different communities whose norms they are not familiar with by exposing in-group norms [9] and exposing information that is desirable across different communities.

### Managing Tensions between Goals and Social Needs

Using Instagram both for tracking one's own health behaviors and for engaging others creates a tension between personal tracking goals and social goals. This tension can cause people to adjust their behaviors to reconcile the two goals. Sometimes this adjustment leads to behaviors that are both better for the user's health and more engaging for others, but sometimes people prioritize one goal over the other, even to the detriment of their own health behaviors.

#### Tracking change when supporting social needs

Membership in the Instagram community influenced how our participants collected information. Previous research has also found that participants wanted to post interesting rather than routine content [41,43], including photos [5]. Findings in other online communities [43] and family journaling [25] also show that people strove to engage and inform their audience with the content they posted, even when this conflicted with personal goals.

Our findings emphasize an underlying conflict in this preference: the desire to keep an Instagram feed interesting and engaging poses a challenge to users who want to record their day-to-day meals. Because of this conflict, all but two participants created separate accounts to prevent over-sharing. To maintain an interesting feed, some participants also lapsed in tracking, which in turn limited their ability to review and reflect on past behavior, such as preventing people from identifying personal antecedents of a negative outcome (e.g., undesired weight gain). One design opportunity could be to support different content lifespan for sharing and reflection. For example, systems could support ephemeral sharing, similar to Instagram Stories, to fulfill people's social needs while maintaining the content privately for future reflection. Designers of tracking systems should also consider supporting reflection according to what people try to learn from their or other people's behavior. For example, when people seek inspiration from past behavior, systems could highlight weeks when the user ate healthy or diversified meals, even given an incomplete record. When reflecting to understand personal behavior, the system could highlight the weeks most representative of food patterns and emphasize when the data might be incomplete.

#### Behavior change when supporting social needs

The conflict between Instagram norms and someone's personal health goals also affects user behaviors, in positive and negative ways. In some cases, social norms influenced participants to act in ways more consistent with their own health goals. For instance, some participants mentioned eating fewer snacks (I12), more vegetables (I03, I05), or more diverse food (I10, I15) because they did not want to disappoint their followers and wanted to stay accountable to them. Their healthy behavior choices were often reinforced by reading other people's posts to learn healthy eating strategies and by receiving feedback from followers. Similar to Community Mosaic users, our participants also made healthier choices so they would not have and then share experiences that might negatively influence others [44].

However, in other cases social pressure made participants less likely to engage in healthy behaviors: I01 swam less frequently because the workout was less accessible to her followers. Increases in negative behaviors and outcomes have occurred in other Instagram communities, such as depression in eating disorder recovery communities [7]. Content can also influence people who run across the posts but are not part of the community, such as people who find pro-anorexia content by searching for fitness or health-related hashtags [45]. As suggested by research on depression online [10], designers or facilitators of online communities should consider creating awareness by proposing community norms for positive behavior. Systems can promote this awareness by supporting people to reflect on their long-term trend in relation to a community's overall behavior. Systems can also involve health experts to moderate negative posts or promote educational material to increase awareness of positive behavior.

### Appropriating Multiple Tools for Social Personal Tracking

Munson et al. [39] describe how repurposing social media platforms for health can be a first-order approximation —a “*tractable solution that partially solves specific problems with known trade-offs*” [1]— that can help individuals attempting to manage different aspects of their health. Using Instagram as a social, photo-based food journal, is one such approximation: it provides features to support food photo collection, reflection on old posts, and finding community support. Instagram was not designed for personal tracking, and does not include common self-tracking features, such as summaries designed to support reflection. As Instagram was not designed to support a focus on healthy eating, it also lacks features to support recording or sharing detailed food information or recipes. The lack of these features might be challenging for people who seek to appropriate Instagram as a personal food tracking system.

However, our findings suggest that participants with varied health goals could appropriate Instagram's features to fit with their needs. Some participants would glance chronologically through the color and content of food photos to find trends and awareness. Others felt more engaged with their tracking without entering all the details. Some of Instagram's flexibility derives from it not being designed for food tracking, and so it does not impose a designer's notion of how people should track food. This allows people to use the photos and text fields as needed to meet their goals.

People also use Instagram alongside other tools to support particular tracking needs, such as I15 who used YouFood for detailed food tracking while also posting photos to Instagram for social support. I05 also use Instagram photos as a reminder of what she had eaten for the day and then used MyFitnessPal for calorie counting. While participants often found tracking different information using several apps useful, they also discussed a need to integrate information from several sources to help them reflect on their holistic health goals. Helping users easily integrate additional information with the right level of detail into their food posts could support their reflection on their behaviors in more detail. Future systems should also consider allowing importing and exporting data from different tools with customized levels of disclosure. In this way, users can select tools that best fit their needs.

## Limitations and Future Work

We searched for users using #fooddiary or #foodjournal at various times of day to recruit participants across different time zones who were posting food photos at different times of day. However, we only encountered one male in over 200 Instagram users we contacted, who did not respond to our interview invitation. We specifically looked for males in snowball sampling. Participants also commented on male scarcity. As a result, participants were all female and 18 to 34 years old, leaving us unable to describe experiences of Instagram users more broadly.

We were also only able to recruit participants who were actively using Instagram to support healthy eating behavior, because hashtag searching on Instagram only shows results from the most current hashtag use. Therefore, although our study includes participants who had previously lapsed, it does not describe the experiences of people who abandoned food tracking on Instagram.

While we strove to understand a range of uses and experiences, including participants with varied numbers of followers and in different stages of pursuing healthy eating, we did not collect socioeconomic status (e.g., education or income). Future research should examine the experiences of people of diverse socioeconomic status. In addition, while our participants had diverse goals and follower counts, our sample size was small. Future, larger-scale research is needed to provide nuanced insight into differences between user groups.

## Conclusion

Building on prior knowledge of how people appropriate social media for the pursuit of health goals, we describe lived experiences of personal food tracking in a social environment. We use the lived informatics model to describe how people's tracking activities were shaped by their intent to receive and provide social support on Instagram. Goals of managing one's impression, receiving and providing social benefits, and keeping track of behavior are intertwined and influence each other.

Designers wanting to support personal tracking in social contexts should consider features that help users pursue social benefits. For example, systems can help users access communities for specific health goals and diverse exploration as well as providing strategies to navigate through different communities. Also, systems can support users in integrating information from different apps that are better fit to various aspects of their health goals.

For personal informatics researchers, the personal informatics models should be further developed to account for social influence. Researchers should reflect on how social needs affect people's decisions about and behavior surrounding the acts of selecting tools, collecting, integrating, and reflecting on data, and lapsing and resuming.

## Figures and Tables

**Table 1:** Interview participant demographics, health goals, and community affiliations

Participant Number	Age	Instagram Experience (months)	Food Posting Experience (months)	Number of Followers	Number Following	Health Goals	Self-Identified Community	Occupation
I01	25-34	> 12	6	198	81	Conscious eating	None	N/A
I02	25-34	> 12	6	462	857	Ketogenic diet	Keto Diet	N/A
I03	18-24	> 12	6	362	536	Eating disorder recovery	None	Student
I04	18-24	> 12	6-12	260	66	Healthy cooking and eating	None	Student
I05	25-34	6-12	6-12	377	323	Weight loss	None	Video games
I06	25-34	6	6	323	415	Weight loss	Weight Watchers	Stay-home Mom
I07	25-34	6-12	6-12	218	224	Weight loss	None	Homemaker
I08	18-24	> 12	6-12	9721	1377	Healthy eating and fitness	Bikini Body Guide	Eating disorder center worker
I09	18-24	> 12	6-12	1991	388	Weight loss	MyFitnessPal	Caretaker in children's home
I10	25-34	6-12	6	3345	133	Weight loss	Weight Watchers	N/A
I11	25-34	> 12	> 12	3005	1081	Ketogenic Diet	Keto Diet	YouTube content creator
I12	25-34	> 12	> 12	98	158	Conscious Eating	None	Student
I13	25-34	> 12	6-12	1066	548	Fitness	Bikini Body Guide	Victim advocate
I14	18-24	> 12	> 12	1276	673	Fitness	Bikini Body Guide	Marketing
I15	25-34	> 12	> 12	6646	144	Weight loss	Weight Watchers	N/A
I16	25-34	> 12	6-12	3455	288	Weight loss	Weight Watchers	Event planner & space coordinator
